# Response Evaluation in Patients with Peritoneal Metastasis Treated with Pressurized IntraPeritoneal Aerosol Chemotherapy (PIPAC)

**DOI:** 10.3390/jcm12041289

**Published:** 2023-02-06

**Authors:** Signe Roensholdt, Sönke Detlefsen, Michael Bau Mortensen, Martin Graversen

**Affiliations:** 1 Odense PIPAC Center, Odense University Hospital, J.B. Winsloews Vej 4, 5000 Odense, Denmark; 2 Department of Oncology, Odense University Hospital, J.B. Winsloews Vej 4, 5000 Odense, Denmark; 3 Department of Pathology, Odense University Hospital, J.B. Winsloews Vej 15, 5000 Odense, Denmark; 4 Odense Pancreas Center (OPAC), Odense University Hospital, J.B. Winsloews Vej 4, 5000 Odense, Denmark; 5 Department of Clinical Research, Faculty of Health Sciences, University of Southern Denmark, J.B. Winsloews Vej 19, 5000 Odense, Denmark; 6 Department of Surgery, Odense University Hospital, J.B. Winsloews Vej 4, 5000 Odense, Denmark

**Keywords:** pressurized intraperitoneal aerosol chemotherapy (PIPAC), peritoneal metastasis, intraoperative chemotherapy, response, peritoneal regression grading score (PRGS), cytology

## Abstract

Pressurized intraperitoneal aerosol chemotherapy (PIPAC) directed therapy emerged as a treatment of peritoneal metastasis (PM) a decade ago. The response assessment of PIPAC is not uniform. This narrative review describes non-invasive and invasive methods for response evaluation of PIPAC and summarizes their current status. PubMed and clinicaltrials.gov were searched for eligible publications, and data were reported on an intention-to-treat basis. The peritoneal regression grading score (PRGS) showed a response in 18–58% of patients after two PIPACs. Five studies showed a cytological response in ascites or peritoneal lavage fluid in 6–15% of the patients. The proportion of patients with malignant cytology decreased between the first and third PIPAC. A computed tomography showed stable or regressive disease following PIPAC in 15–78% of patients. The peritoneal cancer index was mainly used as a demographic variable, but prospective studies reported a response to treatment in 57–72% of patients. The role of serum biomarkers of cancer or inflammation in the selection of candidates for and responders to PIPAC is not fully evaluated. In conclusion, response evaluation after PIPAC in patients with PM remains difficult, but PRGS seems to be the most promising response evaluation modality.

## 1. Introduction

Pressurized intraperitoneal aerosol chemotherapy (PIPAC) directed treatment of peritoneal metastasis (PM) has evolved over the last decade, but results from randomized controlled trials have not been published yet [1,2]. There is a high degree of consensus on the indications, technical aspects, safety, and completion of PIPAC. However, one of the least consensual topics is response evaluation, which may hamper the comparability of future studies [3]. It is essential to have one or several validated, reproducible, objective real-time response monitoring methods available when introducing new oncological treatments. The same applies for new surgical techniques that should follow the clinical steps of the IDEAL framework (idea, development, exploration, assessment, and long-term studies) [4]. PIPAC combines antineoplastic treatment and minimally invasive surgery. Therefore, readily available methods of response evaluation with prognostic information are especially important in this setting where alterations of treatment parameters are continuously being investigated (e.g., diffusion time, electrostatic precipitation, antineoplastic agents, formulations, or doses). 

The limitations of traditional response evaluation methods, such as the radiological response evaluation criteria in solid tumors (RECIST) are well known, but they are still used and reported in several published and ongoing studies [5,6]. Treatment response is often presented in general variables such as median overall survival, progression free survival, peritoneal regression grading score (PRGS), peritoneal cancer index (PCI), eligibility for radical surgery after PIPAC, and quality of life (QoL) [7]. Despite their importance, these parameters are probably not attributable to the isolated effect of PIPAC, but to the combined treatment and patient care efforts. Several reviews have been published on PIPAC, but some do not discuss response evaluation, whereas others report on histological response evaluation only [1,8]. They do not touch upon the lack of non-invasive procedures for objective and reliable response evaluation. Two of the most cited systematic reviews acknowledged limitations regarding the non-invasive evaluation of patients. They urged for the invention of new methods for evaluating response to PIPAC and a standardization of response evaluation methods [9,10]. 

The inflammatory tumor microenvironment is a well-established characterization of carcinogenesis. The host inflammatory response anticipates tumor genesis, tumor invasion, metastasis, and immune surveillance by a pro-cancerous microenvironment. Immune surveillance seems to be particularly poor in ovarian cancer, a frequent origin of PM [11]. It may be hypothesized that biomarkers of the inflammatory burden (e.g., neutrophils, lymphocytes, and the interleukin-6 sensitive acute phase C-reactive protein (CRP)) can predict treatment response. These are easily accessible and analyzed from peripheral blood samples. Several studies have shown that the systemic inflammatory burden in various advanced, non-resectable cancers, evaluated by CRP, albumin, and neutrophil-to-lymphocyte ratio (NLR), has an independent prognostic value [12]. The inclusion of such blood- or tissue-based biomarkers as part of future response evaluation modalities has not been suggested by recent reviews, and a classification and assessment of the performance of different methods of response evaluation currently used to monitor the effect of PIPAC are also lacking.

This review assessed methods of response evaluation in patients with non-resectable PM from various primary tumors treated with PIPAC or electrostatic precipitation PIPAC (ePIPAC). It also investigated whether other variables, such as blood-based biomarkers, may be used to distinguish responders from non-responders to PIPAC treatment.

## 2. Materials and Methods

This is a narrative literature review that investigated methods of response evaluation (excluding survival rates) in non-resectable patients with PM treated with PIPAC. We searched PubMed and clinicaltrials.gov for eligible publications or protocols from 2011 to January 2023. Eligible publications and protocols had to be in the English language and describe a response to PIPAC or ePIPAC by tumor/inflammatory biomarkers, radiology, PCI, PRGS, or cytology. We omitted case reports of less than five patients.

### 2.1. Definition and Classification of Response Evaluation Modalities 

We divided response evaluation modalities into non-invasive and invasive procedures. Results from non-invasive procedures may be available both during initial patient selection and as a screening tool during treatment. The invasive procedures focus solely on treatment response. The PCI score may be obtained both as a non-invasive and invasive staging or response evaluation modality since it can be based on both radiology, surgery (visual inspection), and histology reports. Herein, it is discussed solely among the invasive procedures to avoid repetitions. Composite endpoints are valid for both non-invasive and invasive response evaluation. Data were extracted based on the intention to treat analysis. 

### 2.2. Non-Invasive Procedures

Non-invasive procedures included analysis of serum biomarkers of tumor or inflammation, and radiology. 

### 2.3. Invasive Procedures

Invasive procedures included laparoscopic mapping of PM, peritoneal biopsies analyzed according to the peritoneal regression grading score, and ascites/peritoneal lavage fluid (PLF) sampling for cytological and/or molecular analyses. 

## 3. Results

### 3.1. Non-Invasive Procedures

#### Tumor Related Serum Biomarkers

The use of a specific blood-based biomarker to predict local tumor response was reported early in the era of PIPAC. A multivariate regression analysis in a small study on women with ovarian cancer found that serum cancer antigen 125 (CA-125) was unable to predict a local tumor response [13]. This finding agreed with results of a larger study from the same research group [14]. Carcinoembryonic antigen (CEA) levels were used for tumor response evaluation in a small phase II study on unresectable colorectal PM treated by PIPAC. A significant histological response according to PRGS was noted between baseline and the last PIPAC, but CEA levels did not change [6,15]. A similar study with bidirectional treatment and ePIPAC that uses CEA as part of the response evaluation is ongoing [16]. Index CEA and CA-125 levels were also measured in a phase II trial on gastric cancer patients, but they were not used for response assessment. [17]. Another phase II trial on different primary tumors showed a decrease in the median CA-125, CA 19-9, and CEA after the second and third PIPAC in those patients who had a radiological response to treatment [18]. Serum tumor markers were also measured in other PIPAC studies, but were not specifically correlated to the PRGS score [19,20,21]. Interestingly, a recent international survey on PIPAC revealed that only half of the surveyed (expert) centers used tumor markers for assessment, whereas all centers performed a detailed radiological evaluation [3].

### 3.2. Biomarkers of Inflammation

A recent study on the pre-procedure immunonutritional status of patients undergoing PIPAC showed no difference in blood test scores (NLR, prognostic nutritional index (PNI), and platelet-to-lymphocyte ratio (PLR)) between responders and non-responders according to PRGS [22]. A low index PNI (cut off value of 36.5) was significantly associated with a worse overall survival (hazard ratio 2.41, 95% CI 1.08–5.46) [22]. As outlined by the authors, the small and heterogenous study cohort and lack of longitudinal data sets may explain the lack of a significant correlation to the PRGS response. 

The cachexia–anorexia syndrome (CAS) is unfortunately prominent in patients with PM, and CAS was found to be predictive of overall survival in patients with PM from gastrointestinal tumors [23]. A retrospective longitudinal cohort analysis of the nutritional status of women with PM from (primarily) ovarian cancer treated with PIPAC showed that these patients were in a chronic state of inflammation. Although the treatment seemed to stabilize the deterioration of the nutritional status, none of the investigated parameters (including CRP, albumin, and hemoglobin) were able to predict CAS deterioration [24]. 

### 3.3. Radiology

Nine studies on PIPAC treatment of patients with PM report the radiological response to treatment (Table 1). Four retrospective studies with heterogeneous study populations reported the radiological response at different time points [25,26,27,28]. These studies showed a radiological response after PIPAC in 15–78% of the patients. Five small prospective studies demonstrated radiological response to PIPAC in 22–62% of patients with colorectal, ovarian, gastric, or miscellaneous primary tumors at different timepoints [6,17,18,21,29]. 

According to a published study protocol, the performance of gadolinium enhanced diffusion weighted magnetic resonance imaging (MRI) before and after PIPAC treatment is currently being investigated, but no data have been presented yet and the utility of MRI for response evaluation of PIPAC is still unknown [30]. 

The performance of fluorodeoxyglucose positron emission tomography (FDG PET) combined with a computed tomography (CT) before and during PIPAC is largely unknown. De Simeone et al. evaluated treatment response according to PET response criteria in solid tumors (PERCIST, version 1.1) after two PIPACs, in patients with different primary tumors [18,31]. They compared FDG PET/CT response to a baseline CT scan and showed partial response or stable disease in 13 patients (20%, intention to treat), and progressive disease in 11 patients (17%). 

### 3.4. Invasive Procedures

#### 3.4.1. Peritoneal Cancer Index (PCI)

A PCI score based on radiologic or laparoscopic investigations may be reported prior to PIPAC (index PCI) and during or after repeated PIPAC procedures. Index PCI was mentioned and measured in 17 prospective and more than 30 retrospective studies [6,13,17,21,29,32,33,34,35,36,37,38,39,40,41,42,43]. Median (or mean) index PCI was 15 (range 10–29) in prospective studies, and some reported the PCI score after a varying number of PIPACs [6,13,17,29,35,36,38,39]. Stable or decreased PCI was noted in all studies, and the reported decrease in PCI was seen in 57–72% of the patients. A small retrospective study reported a 77% increase in PCI after PIPAC, whereas the remaining retrospective studies reported stable or decreased PCI [44]. A patient-based comparison between radiological, macroscopic, and cytological/histological response has been attempted, but data are too limited to allow any firm conclusion [6]. 

#### 3.4.2. Peritoneal Regression Grading Score (PRGS)

A histological response to PIPAC has been reported since its introduction a decade ago [45,46]. The first studies used different variants of the tumor regression grading score until a standardized histological regression grading system for PM was proposed in 2016—the four-tiered PRGS [15,47]. The PRGS evaluates peritoneal punch biopsies from the parietal peritoneum of all four abdominal quadrants (if possible). The proposed scores were defined as follows: PRGS 1, Complete histological response; PRGS 2, Major histological response; PRGS 3, Minor histological response; and PRGS 4, No response. The maximum and mean PRGS should be given in the pathology report, and a complete response to treatment should include a negative PLF. In 2019, it was shown that the PRGS is reproducible with substantial interobserver agreement regarding mean PRGS and moderate to substantial agreement regarding maximum PRGS per quadrant biopsy set [48]. The intraobserver variability was excellent. Methods to improve the reproducibility of the PRGS have been investigated in a study including a total of 662 digitalized H&E- and immunohistochemically (IHC) stained slides from 331 quadrant biopsies. The use of IHC improved reproducibility, particularly between less experienced raters, and should be considered at centers implementing a new PIPAC program [49].

The site of biopsy for PRGS assessment may vary between different PIPAC centers. According to a recent survey, 27% of the centers take biopsies from the same sites during repeated treatment (in some centers marked by metal clips), whereas 71% take biopsies from alternate sites [50]. It has been debated whether biopsies taken at the same site could lead to overestimation of treatment response due to scar tissue arising from the first biopsy procedure or from the clips marking. Fallah et al. investigated this by taking a biopsy from the PM with the worst visible malignant features, in addition to the four standard quadrants biopsies. They found no overestimation of regression in repeated biopsies from the same clips marked PM elements according to PRGS [42]. 

The visceral peritoneum is usually not biopsied due to the risk of organ perforation or bleeding. In addition, “non-access” to PM, due to adhesions within the peritoneal cavity, can make it impossible to take biopsies. Consequently, the PRGS cannot be assessed in those situations. 

Twenty-one studies (nine prospective and twelve retrospective) have investigated the use of PRGS as a response evaluation modality after PIPAC (Table 2). In one prospective study on patients with gastric cancer, baseline PRGS scores from PIPAC 1 were not available, but four patients (13%) had a complete response, and five (16%) had a major response at the second PIPAC [37]. Five prospective studies on PM from various primary tumors showed a stable or improved mean PRGS in 18–51% of the patients [18,32,51]. The prospective study on ePIPAC, with only one minute of diffusion time, was stopped due to inferiority compared to standard PIPAC at the second interim analysis [51]. Interestingly, Benzerdjeb et al. showed that only 19% of their patients responded according to the mean PRGS, while 81% responded if measured by the highest value of PRGS at the third PIPAC [34]. One retrospective study in patients with PM from appendiceal cancer showed a complete or major response in 22% of the patients at the third PIPAC [25]. Another retrospective study in patients with PM from colorectal cancer was compromised by discontinued treatment or missing data in 75% of its patients, but showed a major or complete response in 3%, and minor or no response in 7% at the third PIPAC [52]. This study showed no prognostic value of PRGS, which could be due to the limited sample size and missing data. Taibi et al. compared feasibility and outcomes of patients with PM from colorectal cancer treated with oxaliplatin based PIPAC, with or without intraoperative intravenous 5-fluorouracil and leucovorin. They detected a major or complete response in 33% and 22% of the patients at the third PIPAC, while none and 3%, respectively, had no response to treatment [53]. In a retrospective study on patients with gastric cancer, baseline PRGS were not available, but a major or complete response was seen in 27 (19%) and a minor or no response in 9 (6%) patients at the third PIPAC [54]. The authors concluded that the completion of more than two PIPACs had prognostic value, while PRGS did not. Again only 37/144 (26%) of the patients had three PIPACs, which must attenuate the interpretation of these findings. One retrospective study evaluated the response in patients with malignant peritoneal mesothelioma and found a significant reduction in the mean PRGS from the first to the third PIPAC (mean PRGS 3.0 to mean PRGS 2.0) [26]. Three retrospective studies evaluated the use of PRGS in PM from various primary tumors [55,56,57]. Kurtz et al. showed stable or improved mean PRGS in 34%, while the histological response data were unavailable in the two other studies. 

Looking at the epigenetic changes, Rezniczek and colleagues examined snap-frozen peritoneal biopsies from women with PM, mainly ovarian and endometrial cancer, treated with PIPAC. They used a panel of 22 mRNAs and immunohistochemistry, and described molecular changes before and after localized chemotherapy in responders and non-responders [58].

**Table 2:** Studies reporting histological response according to the mean Peritoneal Regression Grading Score (mPRGS) after Pressurized IntraPeritoneal Aerosol Chemotherapy (PIPAC).

Article (Author, Journal, Year)	Design	Patients n	Primary Tumor	Patients Evaluated, n	PIPACs before Evaluation	Stable or Improved mPRGS n (%)	Worsened mPRGS n (%)
Khomyakov, Pleura Peritoneum 2016 [37]	Pro	31	Gastric	15	1	NS	NS
Ellebaek, Clin Exp Metastasis 2020 [43]	Pro	20	Gastric	10	2	6 (30)	4 (20)
Ellebaek, Pleura Peritoneum 2020 [41]	Pro	24	Colorectal	15	2	14 (58)	1 (4)
Rovers, Ann Surg Oncol 2021 [6]	Pro	20	Colorectal	13	2	10 (50)	3 (15)
Graversen, Ther Adv Medical Oncol 2018 [32]	Pro	35	Various	27	2	18 (51)	NS
Graversen, Eur J Surg Oncol 2020 [51]	Pro	33	Various	10	2	6 (18)	4 (12)
De Simone, Biomedicines 2020 [18]	Pro	63	Various	22	1 or 2	20 (32)	2 (3)
Willaert, Eur J Surg Oncol 2019 [21]	Pro	52	Various	28	2	21 (40)	7 (13)
Benzerdjeb, Histopathology 2020 [34]	Pro	112	Various	112	2	21 (19)	91 (81)
Somashekhar, Cancers 2022 [25]	Retro	77	Appendiceal	22	2	NS	NS
Tabchouri, Ann Surg Oncol 2021 [52]	Retro	102	Colorectal	27	2	NS	NS
Taibi, Ann Surg Oncol 2022 [53]	Retro	131	Colorectal	59	2	NS	NS
Di Giorgio, Surg Oncol 2020 [44]	Retro	28	Gastric	12	2 or 3	11 (39)	1 (4)
Sindayigaya, Ann Surg Oncol 2022 [54]	Retro	144	Gastric	37	2	NS	NS
Kepenekian. Ann Surg Oncol 2022 [26]	Retro	26	MPM	14	2	NS	NS
Horvath Clin Exp Metastasis 2018 [59]	Retro	12	Pancreatic Biliary	6	1	6 (50)	0
Di Giorgio, Ther Adv Medical Oncol 2020 [60]	Retro	20	Pancreatic biliary	11	1 or 2	11 (55)	0
Nielsen, J Clin Pathol 2021 [61]	Retro	16	Pancreatic	6	2	5 (31)	1(6)
Kurtz, Gastroenterol Res Pract 2018 [55]	Retro	71	Various	36	1	24 (34)	12 (17)
Ceribelli, Surg Endosc 2020 [56]	Retro	43	Various	21	1	NS	NS
Taibi, Ann Surg Oncol 2021 [57]	Retro	69	Various	22	2	NS	NS

MPM: malignant peritoneal mesothelioma, NS: not specified, Pro: prospective, Retro: retrospective.

#### 3.4.3. Cytology of Ascites or Peritoneal Lavage Fluid (PLF)

Cytological evaluation of ascites or PLF is conducted in five of nine active PIPAC centers worldwide according to a recent survey [3]. Five studies in patients with PM from different primary tumors (four prospective) have evaluated the cytological response to treatment [6,25,32,34,51]. They included 328 patients (range 16–112). Three studies used paired data, which showed a conversion from malignant to a non-malignant status in 6–15% of the patients, and a non-malignant to malignant in 3–5% at the third PIPAC [6,32,51]. Two studies showed that 43–54% of the patients had malignant cytology at the first, and 29–45% at the third PIPAC [34,62]. One study of 77 patients with appendiceal cancer and PM did not report the intention to treat data, but 20% of the per protocol population (n = 35) had malignant ascites/PLF at the first PIPAC procedure, and 9% at the third PIPAC [25]. 

## 4. Discussion

This narrative review shed light on currently available methods for response evaluation of PIPAC directed treatment in patients with PM based on non-invasive and invasive methods. Most studies were retrospective in design with small and heterogeneous study populations. The amount of missing data and discontinued treatment were also substantial and illustrated by the difference in included and evaluated patients (Table 1 and Table 2). Most patients were evaluated after three PIPACs, and the histological response according to PRGS has been the most used modality since its introduction in 2016 [15]. A histological response after two PIPACs was shown in 18–58% of all patients across primary tumor entity. Importantly, biopsies from the third PIPAC represented the histological regression after two PIPACs, since biopsies were taken before nebulization of chemotherapeutic agents. Most studies assumed that a decreasing value of PRGS in biopsies from successive PIPACs should be interpreted as a sign of response. However, recent data revealed that a cut-off of mean PRGS 2, or an absolute decrease in mean PRGS of ≥1.0, should be used to reach significant prognostic value of the PRGS [63]. Benzerdjeb et al. suggested the use of a combined positive index (CPI+) in a study where most patients received systemic chemotherapy before PIPAC treatment was initiated. CPI+ was defined as an increase in maximum PRGS from baseline compared to PIPAC 3 and/or malignant PLF or ascites cytology at PIPAC 3. Patients who were CPI+ had a worse overall survival and a worse progression free survival, compared to patients who were CPI−. This supports the use of PRGS in combination with PLF sampling for response evaluation [34]. The PRGS was not found to have independent prognostic value in this study. However, in one third of the cases, only one peritoneal biopsy was obtained at PIPAC 3. The PRGS assessment was performed by two pathologists, and it seems that upfront IHC was not used, even though more than half of all patients had PM from a poorly cohesive gastric cancer, which can be difficult to detect without IHC. Future studies are needed to evaluate the exact role of molecular analyses of peritoneal biopsies and possibly also ascites or PLF in the setting of PIPAC. Such studies should likely also include RNA sequencing, spatial gene expression profiling, and single cell sequencing techniques.

The defining PRGS publication by Solass et al. in 2016 stated that PRGS should be supported by cytological evaluation of ascites or PLF, particularly before concluding the complete response [15]. Interestingly, only five studies have shown data on the cytological response evaluation, and the definition of response was not uniform. PIPAC was able to eliminate cancer cells in 6–15% of the patients, and the proportion of patients with malignant cytology decreased between the first and the third PIPAC. The actual role of cytological evaluation of ascites or PLF remains a challenge. The fundamental problem with conventional cytology in patients with proven PM is a sensitivity of only 0.58 [62]. This sensitivity must be improved by new techniques, such as additional staining or molecular analyses, before any conclusions on the prognostic value of cytology can be drawn. To our knowledge, the first study examining the utility of PLF, or ascites cytology combined with additional techniques for detection of markers of free intraperitoneal tumor cells (FITC) in the PIPAC setting was published in 2019 [62]. Ascites or PLF obtained prior to the first and third PIPAC was analyzed by conventional cytology, protein analysis (carcinoembryonic protein (CEA) and total protein concentration), and PCR (for mRNA of CEA, epithelial cell adhesion molecule (EpCAM) and CA-125). CEA mRNA and EpCAM mRNA were useful as markers of FITC with a high sensitivity and excellent specificity. However, when using the PRGS as a determinant of response, neither cytology, nor CEA protein and CEA and EpCAM mRNA were useful for identification of PIPAC responders. Recently, a study examined the utility of targeted next generation sequencing (NGS) for the detection of cancer-related mutations in peritoneal quadrant biopsies, PLF, or ascites from patients with pancreatic cancer treated with PIPAC [61]. It was possible to identify the expected mutations both before and after PIPAC. It is not known if this approach can be used for treatment response assessment in larger-scale studies.

If curative surgery is considered possible after PIPAC in patients with previous non-resectable PM, this approach should be supported by a negative cytology and vice versa. In the palliative setting, the role of cytology in response assessment should probably include serial molecular investigations and be performed in well-defined cancer cohorts.

The PCI was first suggested by Sugarbaker in 1996, dividing the visceral and parietal peritoneum into 13 regions [64]. The PCI is commonly used as a prognostic indicator of complete cytoreduction in patients with PM, and the prognostic value of the PCI for patients undergoing cytoreductive surgery and adjuvant treatment is substantiated in several studies [65,66,67]. However, the visual discrimination between treatment-induced fibrosis and active PM elements is difficult and hampers response evaluation according to PCI. A recent study found a poor correlation between the surgical and histological PCI based on microscopy of the index regions [68]. An index PCI score was reported in the majority of PIPAC studies. Repeated scores for response evaluation after several PIPACs were more difficult to evaluate due to study design and heterogeneous patient cohorts. Data from prospective studies suggest a decrease in PCI in approximately two of three patients after PIPAC, but these findings were seldom correlated to other response variables such as PRGS. Despite using repeated PCI scores in both published and ongoing trials, many authors consider visual evaluation of the peritoneal surface after PIPAC as being unable to discriminate between treatment induced fibrosis, and active sites of cancer. Therefore, PCI may essentially be used as a demographic variable describing the study population at baseline, and not as a measure of response. 

Radiological staging is essential before and during antineoplastic treatment of cancer patients. This staging is usually based on a CT, FDG-PET, or MRI. In study protocols, treatment must be evaluated by the RECIST criteria [69]. According to these criteria, response should be evaluated by an enlargement or reduction of target lesions determined at baseline. Further, the occurrence of new lesions should be classified as progressive disease. Thus, treatment may lead to a complete or partial response, stable or progressive disease. Unfortunately, the RECIST criteria state that measurable target lesions must be at least 10 millimeters, and that ascites and palpable abdominal masses (without a CT correlate) are truly non-measurable lesions. Patients with PM treated with PIPAC usually have a miliary distribution of small (<10 mm) elements on the peritoneal surface or a general thickening of the peritoneum, which both may not be evaluated according to RECIST. A radiological stable or responsive disease after two or three PIPACs, according to the RECIST criteria, was seen in 15–78% of all patients. Unfortunately, some studies did not reveal the number of PIPACs before radiological response evaluation. Crucially, most studies did not use the radiological response evaluation at all, arguably due to the described limitations including lack of target lesions according to RECIST [69]. Despite these obvious limitations, RECIST is still being used in ongoing and planned PIPAC trials. The use of fluorodeoxyglucose positron emission tomography (FDG-PET)/CT as a staging or surveillance tool has increased across different primary tumors such as colorectal, esophago-gastric, and bile duct cancers in recent years [70,71,72]. Obviously, the utility and prognostic value of PET/CT must be further evaluated. The recently introduced Ga-68-labelled fibroblast activation protein inhibitor (^68^Ga-FAPI) PET/CT may detect PM more accurately than FDG PET/CT and should be considered in future studies of response to PIPAC treatment [73].

The selection of patients and identification of responders to PIPAC based on blood samples including biomarkers of cancer and inflammation is intriguing. However, scores based on one or several blood-based biomarkers may not directly reflect local response to PIPAC. Local response depends on the reaction to both local and systemic chemotherapy, the individual intraperitoneal (micro)environment, and overall tumor burden. Nevertheless, such scores may provide easy, available prognostic information that could help identify eligible patients for or responders to PIPAC. Theoretically, prognostic, and predictive scores based on blood tests may include tumor related biomarkers such as CA 19-9 or CA-125, nucleic acid-based markers (e.g., circulating tumor DNA or circulating RNA), inflammatory, hematological and organ related markers (e.g., C-reactive protein, hemoglobin, albumin), or a combination of several. The pre-treatment levels of inflammatory biomarkers are probably of most relevance and interest, since they are not influenced by repeated procedure induced intraperitoneal inflammation. However, in light of the recent discovery of the prognostic relevance of PRGS, during the first and third PIPAC procedure, it would be of great interest to look into the correlation between PRGS and serial analyses of simple blood biomarkers in large and uniform PM populations [63]. Still, there are no specific data on the use of biomarkers as composite endpoints for response evaluation, reflecting the difficult interpretation of such surrogate markers. Therefore, these entities are still premature, and more data are needed before defining their clinical impact. 

Despite a meticulous appraisal of the current literature on response evaluation during PIPAC, this review holds limitations. We evaluated PIPAC as an overall treatment, even though PIPAC is merely a drug delivery system. The aerosolized chemotherapeutic agent that holds antineoplastic activity is the actual treatment. Further subgroup analysis based on chemotherapeutic agents (oxaliplatin or cisplatin/doxorubicin) was not appealing due to the small and heterogeneous study populations. As opposed to a systematic review, the search strategy was not shown, which could have conferred selection bias and opacity. Further, we only searched PubMed and clinicaltrials.gov, and no other databases such as Embase. This review was conceptualized to give a status of response evaluation during PIPAC, and therefore did not describe each modality’s accuracy, applicability, or prognostic value in other treatments. The authors chose to focus on the histological response according to PRGS, which omitted a few papers that used other histology-based response evaluation methods, which should be mentioned as a limitation. This strategy uniformed, but potentially also excluded, important data. 

In conclusion, response evaluation after PIPAC in patients with PM remains difficult. Currently, histology-based response evaluation, for example using the PRGS, seems to be the most promising response evaluation modality. Based on large and homogeneous study populations, future studies must compare immediate available response data to survival to decode their final prognostic value. 

## Figures and Tables

**Table 1:** Studies reporting radiological response according to Response Evaluation Criteria in Solid Tumors (RECIST) after Pressurized IntraPeritoneal Aerosol Chemotherapy (PIPAC).

Article (Author, Journal, Year)	Design	No. Patients	Primary Tumor	Patients Evaluated, n	No. PIPACs before Evaluation	CR/PR/SD, n (%)	PD, n (%)
Rovers, Ann Surg Oncol 2021 [6]	Pro	20	Colorectal	13	3	10 (50)	3 (15)
Struller, Ther Adv Med Oncol 2019 [17]	Pro	25	Gastric	25	NS	10 (40)	3 (12)
Tempfer, Gynecol Oncol 2015 [29]	Pro	53	Ovarian	30	3	33 (62)	17 (32)
De Simone, Biomedicines 2020 [18]	Pro	63	Various	40	2	14 (22)	26 (41)
Willaert, Eur J Surg Oncol 2019 [21] *	Pro	48	Various	37	NS	18 (38)	19 (40)
Somashekhar, Cancers 2022 [25]	Retro	77	Appendiceal	35	3	15 (20)	5 (6.4)
Rezniczek, BMC Cancer 2020 [28]	Retro	44	Breast endometrial	22	NS	21 (48)	1 (2)
Kepenekian, Ann Surg Oncol 2022 [26]	Retro	26	MPM	16	3	4 (15)	NS
Girshally, World J Surg Oncol 2016 [27]	Retro	9	Various	9	NS	7 (78)	2 (22)

CR: complete response, MPM: malignant peritoneal mesothelioma, No.: number of patients, NS: not specified, PD: progressive disease, PR: partial response, Pro: prospective, Retro: retrospective, SD: stable disease, * not evaluated according to RECIST.

## Data Availability

Not applicable.
